# Association of CYP2R1 and CYP27B1 genes with the risk of obesity and vitamin D metabolism in Saudi women

**DOI:** 10.1186/s43141-023-00508-7

**Published:** 2023-05-15

**Authors:** Sahar Abdulaziz AlSedairy, Laila Naif Al-Harbi, Manal Abdulaziz Binobead, Jegan Athinarayanan, Shaista Arzoo, Dalia Saade Al-Tamimi, Ghalia Shamlan, Ali Abdullah Alshatwi, Vaiyapuri Subbarayan Periasamy

**Affiliations:** Department of Food Science & Nutrition, College of Food and Agricultural Sciences, King Saud University, Riyadh, 11451 Saudi Arabia

**Keywords:** Vitamin D, Obesity, Genetic variants, Genes, BMI, Saudi

## Abstract

**Background:**

Epigenome, genetic variants, and other environmental factors involved in gene regulation are highly inter-dependent in several chronic diseases, including obesity, cardiovascular disease, and diabetes. The present study aimed at testing the associations and the mechanism involved in silencing of CYP2R1 gene in normal and obese Saudi women patients. Height, weight, BMI, 25-hydroxy vitamin D, parathyroid hormone, glycemic status, and lipid profile (TG, LDL, HDL, and TC) of CYP2R1 were measured in 100 women (31 normal and 69 obese patients).

**Results:**

Our result shows that hypermethylation in site 2 of the CYP2R1 gene with body weight (*p* < 0.004), BMI (*p* < 0.002), waist circumference (*p* < 0.002), total-LDL (*p* < 0.027), total cholesterol (*p* < 0.022), and vitamin D (VD) (close to borderline significance *p* < 0.06) and site 4 of CYP2R1 with LDL (*p* < 0.041) in the four tested sites among normal and obese women was significantly associated. Moreover, we tested five different CpG sites in the CYP27B1 gene where site 5 correlated significantly with VD levels.

**Conclusion:**

Our present study clearly indicates that hypermethylation of specific sites in the CYP2R1 and CYP27B1 genes might regulate gene expression with special reference to the risk of obesity and vitamin D metabolism.

**Supplementary Information:**

The online version contains supplementary material available at 10.1186/s43141-023-00508-7.

## Background

Obesity-associated noncommunicable diseases are complex, multifactorial diseases that are a big challenge in the treatment and management. These diseases have great impacts on the economies of the developed and developing countries. WHO reported that 2.8 million people die each year due to obesity and its associated noncommunicable diseases [29]. The prevalence of obesity cases is exponential in Saudi Arabia for the past few decades [19]. Obesity may occur due to several causative factors including unhealthy food habits, sedentary lifestyles, and genetic and environmental factors. Recent evidence from nutrigenetics and nutrigenomics data has alarmed that the susceptible gene networks are complex and are key players in obesity-linked noncommunicable diseases. Deriving from these data, gene regulation in chronic diseases is highly complex. Specifically, epigenome, genetic variants, and other environmental factors involved in gene regulation are highly inter-dependent. More specifically, genome-wide methylation data analysis clearly indicated that epigenome plays a crucial role in gene regulation [26].

Different meta-analyses clearly indicated that vitamin D deficiency is strongly linked with obesity, diabetes, and cardiovascular diseases [25, 27]. In obesity and vitamin D metabolism-related gene networks, vitamin D receptor (VDR), cytochrome P450 gene family, i.e., CYP2R1, CYP24A1, and CYP27B1, are key players in vitamin D metabolism and interconnected with energy balance, adipocyte physiology, and adipokine secretion. Approximately, 200 genes participate in the pathophysiology of obesity [25, 32]. The gene regulations are still unclear with SNP, microRNA, and epigenomic level [14]. Especially, DNA methylation sites in the promotor region inhibit the binding of specific transcription factors which cause downregulation of specific genes.

Commonly, DNA methylation and genetic variants are linked with downregulation of gene expression. For example, in vivo studies revealed that gene expression of CYP2R1, CYP27A1, CYP27B1, and vitamin D receptor is downregulated due to hyper–methylation in CpG sites [7, 21]. SNPs in CYP2R1 and CYP27B1 genes are strongly associated with abnormal vitamin D metabolism and linked to various pathophysiological conditions [22, 23, 30, 28, 32]. In another study, hypermethylation in the CpG islands of both CYP2R1 and CYP27B genes is susceptible to negative gene regulation [9]. Consequently, epigenetic alterations such as hypermethylation in CYP2R1 and CYP27B1 genes are reported to interfere in the metabolic pathway and decrease vitamin D levels, thereby contributing to genesis of obesity-linked diseases [1]. Gene-specific hypermethylation in the CYP2R1, CYP27B1, and VDR genes was observed in the obesity-induced mice [21].

Parathyroid hormone (PTH) is directly involved in calcium homeostasis. A few studies have indicated that aberrant methylation patterns downregulate CYP2R1, CYP27B1, and VDR genes which are involved in the PTH-dependent calcium metabolism [13]. Besides, CYP2R1 and CYP27B1 are implicated in altered lipid profiles. Mice fed a high-fat diet demonstrated decreased CYP2R1 and CYP27B1 expression [5]. Specifically, CYP2R1 is downregulated in the extrahepatic and liver tissues in the obesity-induced mice model which causes vitamin D deficiency [8]. Likewise, mice fed a high-fat diet evidenced decrease of vitamin D deficiency, whereas expression of CYP1R1, CYP24A1, and CYP27B1 negatively altered lipid profile and increased lipogenesis [6]. Globally, a few meta-analyses have clearly indicated the association between VD metabolism and obesity in different ethnic populations. However, there is no epigenetic data on obesity-related traits and VD metabolism genes in Saudi populations. Therefore, the present study was undertaken to find the correlation of methylation patterns with CYP1R1 and CYP27B1 genes between normal and obese Saudi women.

## Methods

### Samples

The experimental population was 100 Saudi adult females (18 to 60 years) who were selected randomly wherein 31 got assigned to normal weight group [body mass index (BMI; in kg/m^2^) ≤ 25] and 69 patients were assigned the obese group [body mass index (BMI; in kg/m^2^) > 30]. Care was taken to ensure that the patients were not diagnosed with chronic diseases and attended fitness clubs in different areas (north, east, west, and central area) within Riyadh city. Informed consent was obtained from all patients. The study was approved by King Saud University, Saudi Arabia (reference no.: E-19–4028). The study is in concurrence with the policy of the College of Food and Agriculture Sciences Research Centre and within the ethical boundaries of Declaration of Helsinki.

### Anthropometric measurements

Anthropometric parameters such as weight, height, body mass index (BMI), and waist circumference were measured according to our previous report [2].

### Biochemical assessments

Intravenous blood samples were collected after overnight fasting. A part of the blood sample was centrifuged after coagulation, and serum was used for routine biochemical analysis. Another portion of the blood sample was transferred to a heparinized tube for methylation studies and stored at − 80 °C until assays were performed. Biochemical parameters such as fasting serum levels of lipids profile (TG, LDL, HDL, and TC) were measured using an automated chemical analyzer (UDICHEM-300, USA) according to the reference protocols — UI59L, UI41HD, UI 24, etc. Vitamin D and PTH were measured using the cobas e602 analyzer (Roche Diagnostics, Indianapolis, IN, USA).

### DNA methylation analyses by pyrosequencing method

Genomic DNA was isolated from whole blood samples using the Gentra Puregene Blood Kit (Qiagen, Valencia, CA, USA). Purified genomic DNA sample was then bisulfite converted using an EpiTect Bisulfite Kit (Qiagen, USA). Once treated, NaBis-DNA was amplified using PyroMark PCR Kit (Qiagen, USA) with specific primers of CYP2R1 and CYP27B1. DNA methylation levels at CpG sites were assessed using pyrosequencing (PyroMark Q24, Qiagen). All the steps in pyrosequencing assay were practiced according to the manufacturer’s instructions.

### Statistical analysis

Statistical analysis was carried out using the GraphPad Prism 9 software (GraphPad Software, CA, USA) and Microsoft Excel 2007 (Microsoft Corporation, USA). The variables were presented as mean ± standard deviation (SD). Group comparisons were conducted using an independent Student’s *t*-test and Mann–Whitney *U*-test for variables not normally distributed. Correlation between various variables was calculated using Spearman rank correlation coefficient (R) with graphical representations designed using linear regression. The *p*-value was considered significant if less than 0.05.

## Results

**Fig. 1:**
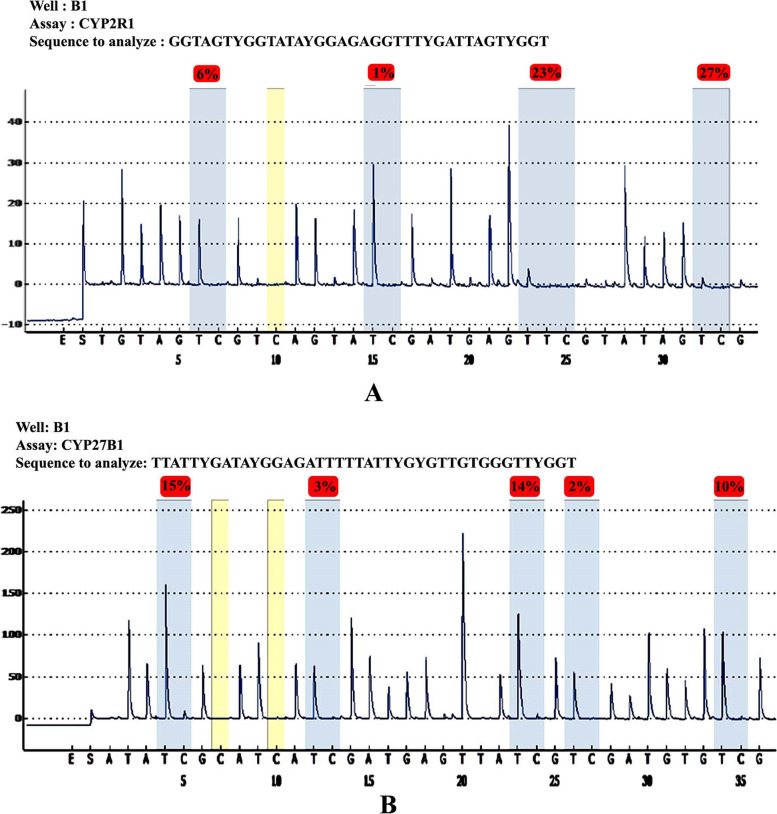
Pyrogram of CYP2R1 methylation sites 1 to 4 (**A**) and pyrogram of CYP27B1 methylation sites 1 to 5 (**B**)

**Fig. 2:**
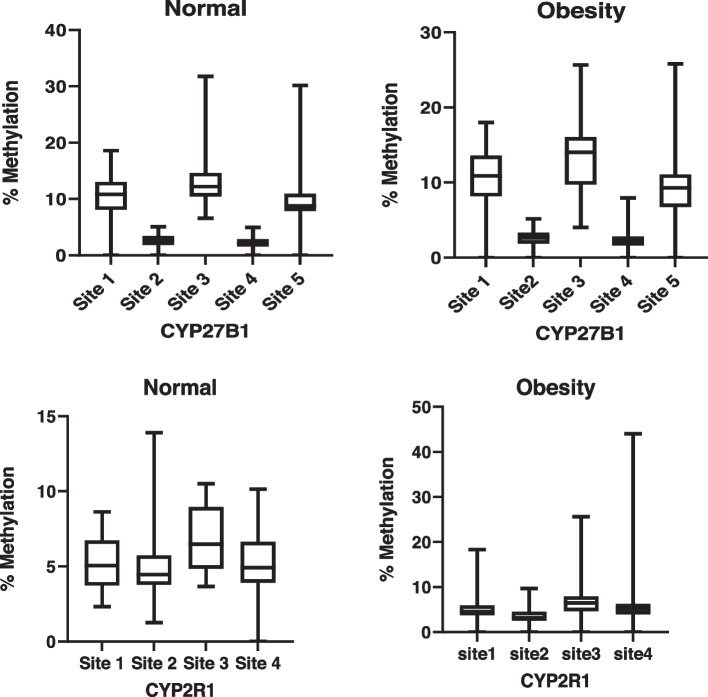
Box plot of median DNA methylation of 5 CpG sites within the promoter of CYP27B1 and 4 CpG site of CYP2R1 genes

**Fig. 3:**
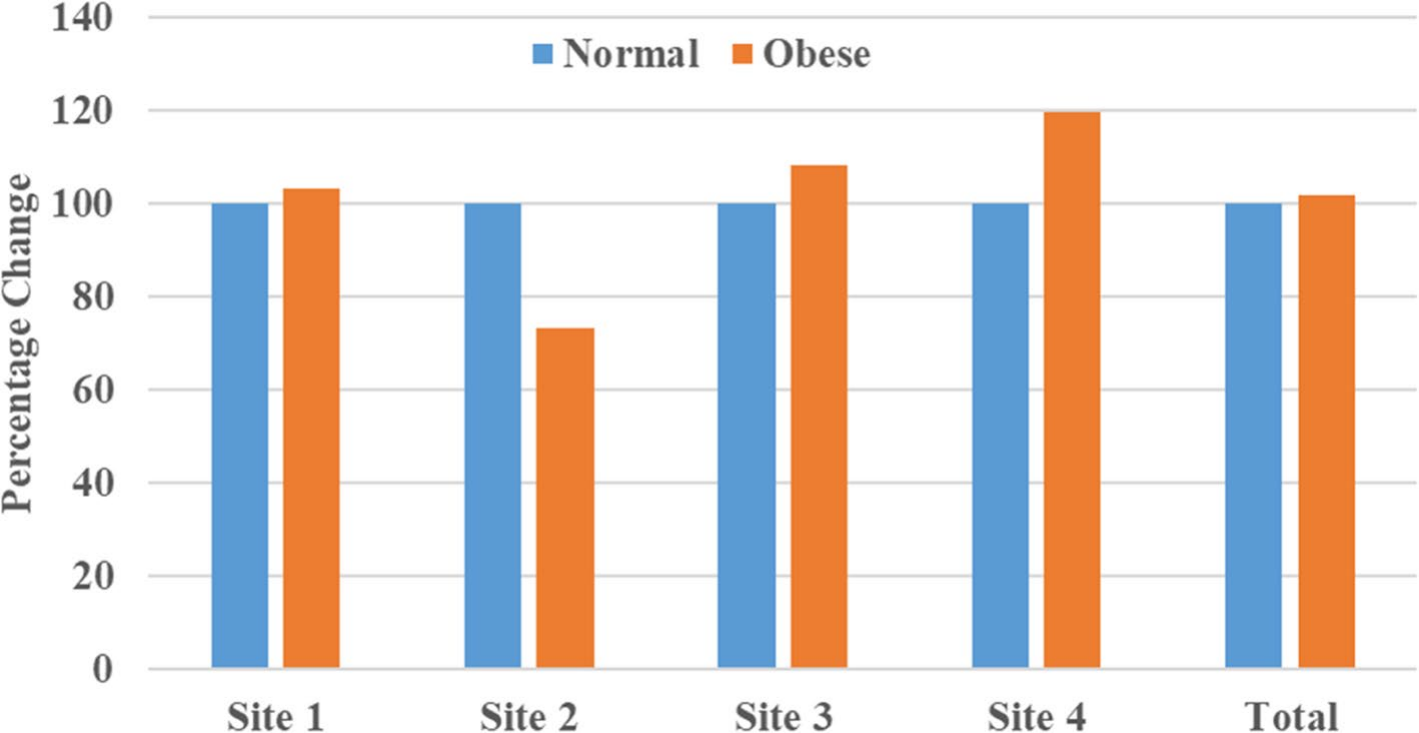
Comparison between total promoter methylation of CYP2R1 of normal and obese samples

In the present study, we analyzed the CYP2R1 and CYP27B1 methylation patterns and their associations with anthropometric parameters, lipid profiles, and vitamin D levels in Saudi obese women. The pyrogram of CYP27B1 and CYP2R1 genes were indicate different CpG sites (Fig. 1 A & B). The box plots show means methylation percentage between samples from normal and obese women for the CYP27B1 and four CpG sites of CYP2R1 genes (Fig. 2). The inside of each box represents the median, and the lower and upper edges of the boxes represent 5 to 45% methylation. The upper and lower lines outside the boxes represent the minimum and maximum values (error bars). Overall, a comparison of different sites of CYP2R1 and CYP27B1 genes of normal vs obese women indicates that there was little change in respect of methylation in obese women compared to the nonobese controls except at site 2 of CYP2R1, i.e., DNA methylation at site 2 of CYP2R1 gene is statistically significant (*p* < 0.001 — Mann–Whitney Test) (Table 1; Fig. 3), and DNA methylation of CYP27B1 gene is not statistically significant. (Supplementary data Table S1 and Figs. S1–S7). Overall, methylation of CpG sites in CYP2R1 and CYP27B1 was analyzed in regard to anthropometric and biochemical parameters. In Spearman correlation analysis, anthropometric parameters such as weight, BMI, waist circunference, plasma, and biochemical parameters, viz., TG, TC, LDL, HDL, VD, and PTH levels, showed a weak negative and positive correlation with CYP2R1 and CYP27B1. As shown in Tables 2 and 3, a positive correlation with site 2 of CYP2R1 with weight (*p* < 0.004), BMI (*p* < 0.002), waist circumference (*p* < 0.002), TC (*p* < 0.022), and LDL (*p* < 0.027) and site 4 with LDL (*p* < 0.041) was significantly correlated (Table 2). CYP27B1 methylation status was not significantly related to anthropometric and biochemical parameters except site 5 with VD (*p* < 0.047) (Table 3).

**Table 1:** Comparison between total promoter methylation of CYP2R1 of normal and obese samples

**Parameters**	**Groups**	***N***	**Min**	**Max**	**Mean ± SD**	**Percent change**	***P*-Value**
Site 1a	Normal	42	2.32	8.63	5.15 ± 1.59	100.00	0.507
	Obese	69	0.00	18.31	5.32 ± 2.95	103.30	
Site 2a	Normal	42	1.26	13.90	4.86 ± 2.11	100.00	0.001
	Obese	69	0.00	9.67	3.56 ± 1.79	73.19	
Site 3a	Normal	42	3.67	10.51	6.82 ± 2.16	100.00	0.841
	Obese	69	0.00	25.60	7.38 ± 4.89	108.23	
Site 4a	Normal	42	0.00	10.14	5.18 ± 2.01	100.00	0.582
	Obese	69	0.00	44.02	6.20 ± 5.91	119.62	
Total^a^	Normal	42	12.15	32.46	22.01 ± 5.84	100.00	0.435
	Obese	69	5.16	58.41	22.46 ± 10.81	102.02	

^a^ Comparison between groups using Mann–Whitney test (nonparametric data)

**Table 2:** Spearman correlation between sites of CYP2R1 methylation with different anthropometric and biochemical parameters

**Parameters (site 1)**	**R (SC)**	**Sig**	**Parameters (site 2)**	**R (SC)**	**Sig**	**Parameters** **(site 3)**	**R (SC)**	**Sig**
Site 1 with wt^	− 0.024	0.803	Site 2 with wt^	0.272^b^	0.004	Site 3 with wt^	0.031	0.749
Site 1 with BMI	− 0.013	0.895	Site 2 with BMI	0.294^b^	0.002	Site 3 with BMI	0.017	0.855
Site 1 with waist	− 0.094	0.326	Site 2with waist	0.289^b^	0.002	Site 3 with waist	0.001	0.996
Site 1 with TC	− 0.050	0.603	Site 2 with TC	− 0.217^a^	0.022	Site 3 with TC	− 0.009	0.927
Site 1 with TG	0.021	0.828	Site 2 with TG	− 0.143	0.135	Site 3 with TG	0.036	0.705
Site 1 with HDL	− 0.026	0.785	Site 2 with HDL	0.034	0.728	Site 3 with HDL	0.135	0.162
Site 1 with LDL	− 0.108	0.262	Site 2 with LDL	− 0.211^a^	0.027	Site 3 with LDL	− 0.102	0.290
Site 1 with VD	0.026	0.807	Site 2 with VD	0.197	0.060	Site 3 with VD	0.108	0.305
Site 1 with PTH	− 0.105	0.277	Site 2 with PTH	− 0.155	0.235	Site 3 with PTH	− 0.007	0.946
**Parameters** **(site 4)**	**R (SC)**	**Sig**	**Parameters (total)**	**R (SC)**	**Sig**			
Site 4 with wt^	0.076	0.431	Total with wt^	− 0.044	0.645			
Site 4 with BMI	0.069	0.474	Total with BMI	− 0.034	0.722			
Site 4 with waist	− 0.042	0.666	Total with waist	− 0.092	0.339			
Site 4 with TC	− 0.103	0.283	Total with TC	− 0.035	0.716			
Site 4 with TG	− 0.063	0.514	Total with TG	− 0.017	0.860			
Site 4 with HDL	0.119	0.218	Total with HDL	0.104	0.280			
Site 4 with LDL	− 0.195^a^	0.041	Total with LDL	− 0.107	0.264			
Site 4 with VD	− 0.056	0.599	Total with VD	0.045	0.668			
Site 4 with PTH	0.097	0.313	Total with PTH	0.014	0.885			

*SC* Spearman correlation, *wt^* weight

^a^ Correlation is significant at the 0.05 level

^b^ Correlation is significant at the 0.01 level

**Table 3:** Spearman correlations between sites of CYP27B1 methylation with different anthropometric and biochemical parameters

**Parameters (site 1)**	**R (SC)**	**Sig**	**Parameters (site 2)**	**R (SC)**	**Sig**	**Parameters (site 3)**	**R (SC)**	**Sig**
Site 1 with wt^	0.116	0.187	Site 2 with wt^	0.103	0.242	Site 3 with wt^	0.123	0.163
Site 1 with BMI	0.061	0.486	Site 2 with BMI	0.095	0.282	Site 3 with BMI	0.095	0.281
Site 1 with waist	0.079	0.374	Site 2 with waist	0.112	0.204	Site 3 with waist	0.100	0.257
Site 1 with TC	0.040	0.649	Site 2 with TC	0.018	0.837	Site 3 with TC	− 0.010	0.908
Site 1 with TG	0.042	0.634	Site 2 with TG	0.051	0.564	Site 3 with TG	0.026	0.769
Site 1 with HDL	− 0.018	0.838	Site 2 with HDL	− 0.044	0.619	Site 3 with HDL	− 0.101	0.255
Site 1 with LDL	− 0.073	0.408	Site 2 with LDL	− 0.043	0.627	Site 3 with LDL	− 0.063	0.476
Site 1 with VD	− 0.060	0.542	Site 2 with VD	− 0.077	0.430	Site 3 with VD	− 0.181	0.062
Site 1 with PTH	0.042	0.639	Site 2 with PTH	0.030	0.741	Site 3 with PTH	0.064	0.475
**Parameters (site 4)**	**R (SC)**	**Sig**	**Parameters (site 5)**	**R (SC)**	**Sig**	**Parameters (rotal)**	**R (SC)**	**Sig**
Site 4 with wt^	0.093	0.292	Site 5 with wt^	0.068	0.443	Total with wt^	0.106	0.229
Site 4 with BMI	0.055	0.530	Site 5 with BMI	0.029	0.742	Total with BMI	0.066	0.454
Site 4 with waist	0.060	0.496	Site 5 with waist	0.022	0.800	Total with waist	0.075	0.396
Site 4 with TC	− 0.037	0.678	Site 5 with TC	− 0.061	0.486	Total with TC	− 0.002	0.984
Site 4 with TG	− 0.015	0.861	Site 5 with TG	0.030	0.733	Total with TG	0.029	0.742
Site 4 with HDL	− 0.059	0.509	Site 5 with HDL	− 0.090	0.313	Total with HDL	− 0.069	0.435
Site 4 with LDL	− 0.053	0.549	Site 5 with LDL	− 0.107	0.226	Total with LDL	− 0.074	0.404
Site 4 with VD	− 0.105	0.284	Site 5 with VD	− 0.192^a^	0.047	Total with VD	− 0.153	0.116
Site 4 with PTH	0.124	0.165	Site 5 with PTH	− 0.045	0.611	Total with PTH	0.047	0.598

*SC* Spearman correlation, *wt^* weight

^a^ Correlation is significant at the 0.05 level

**Fig. 4:**
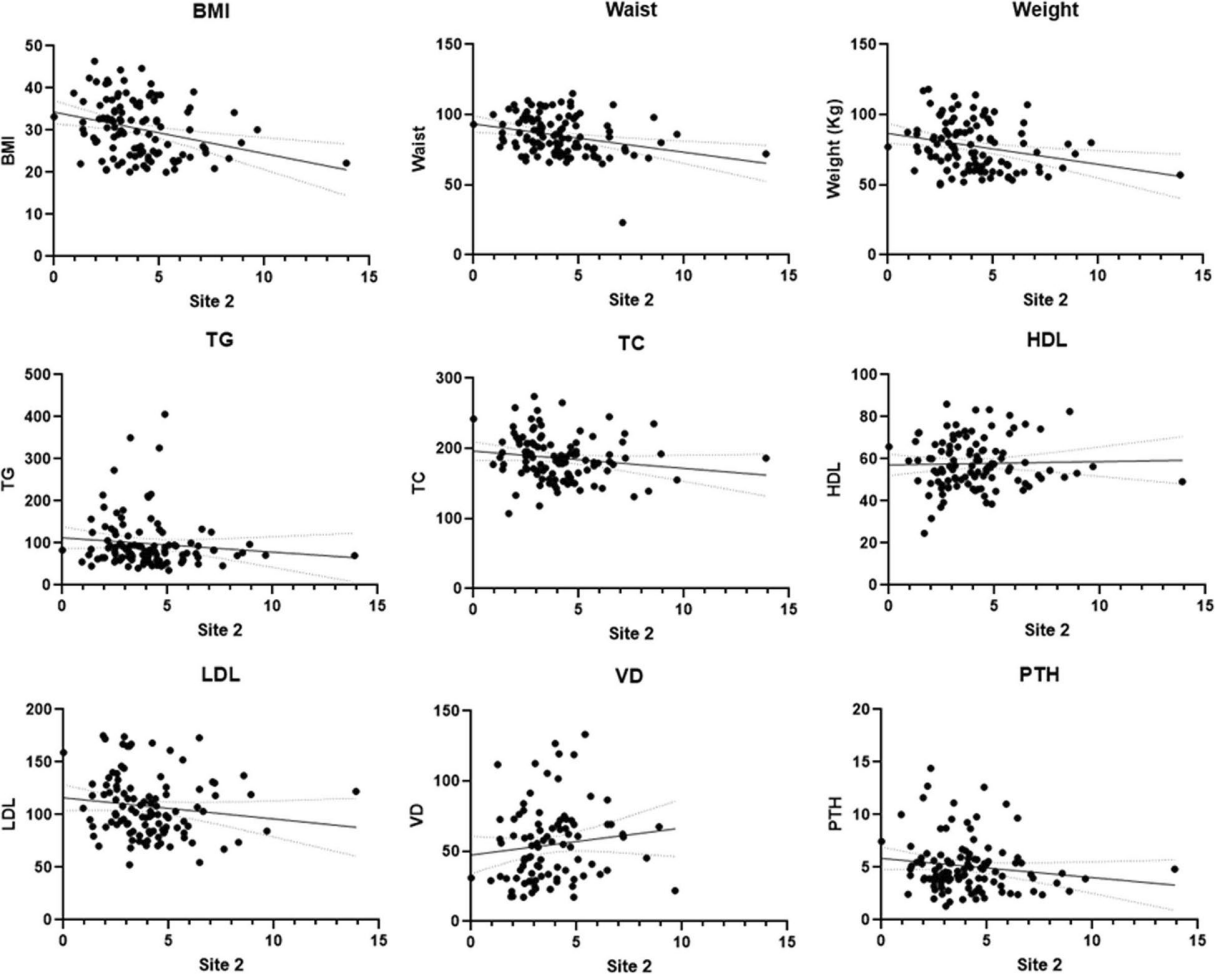
Spearman correlations between site 2 of CYP2R1 methylations with different anthropometric and biochemical parameters

**Fig. 5:**
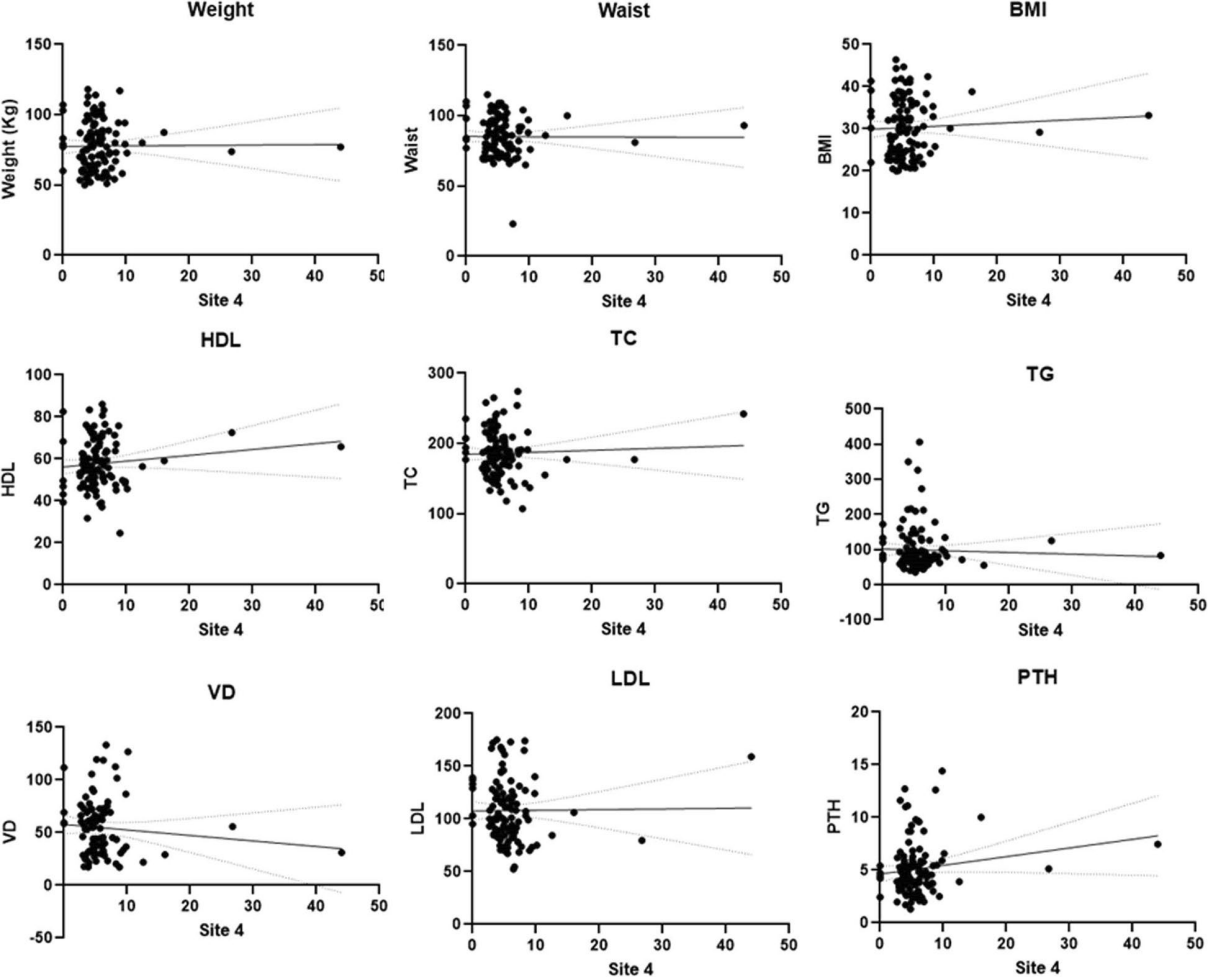
Spearman correlations between site 4 of CYP2R1 methylations with different anthropometric and biochemical parameters

**Fig. 6:**
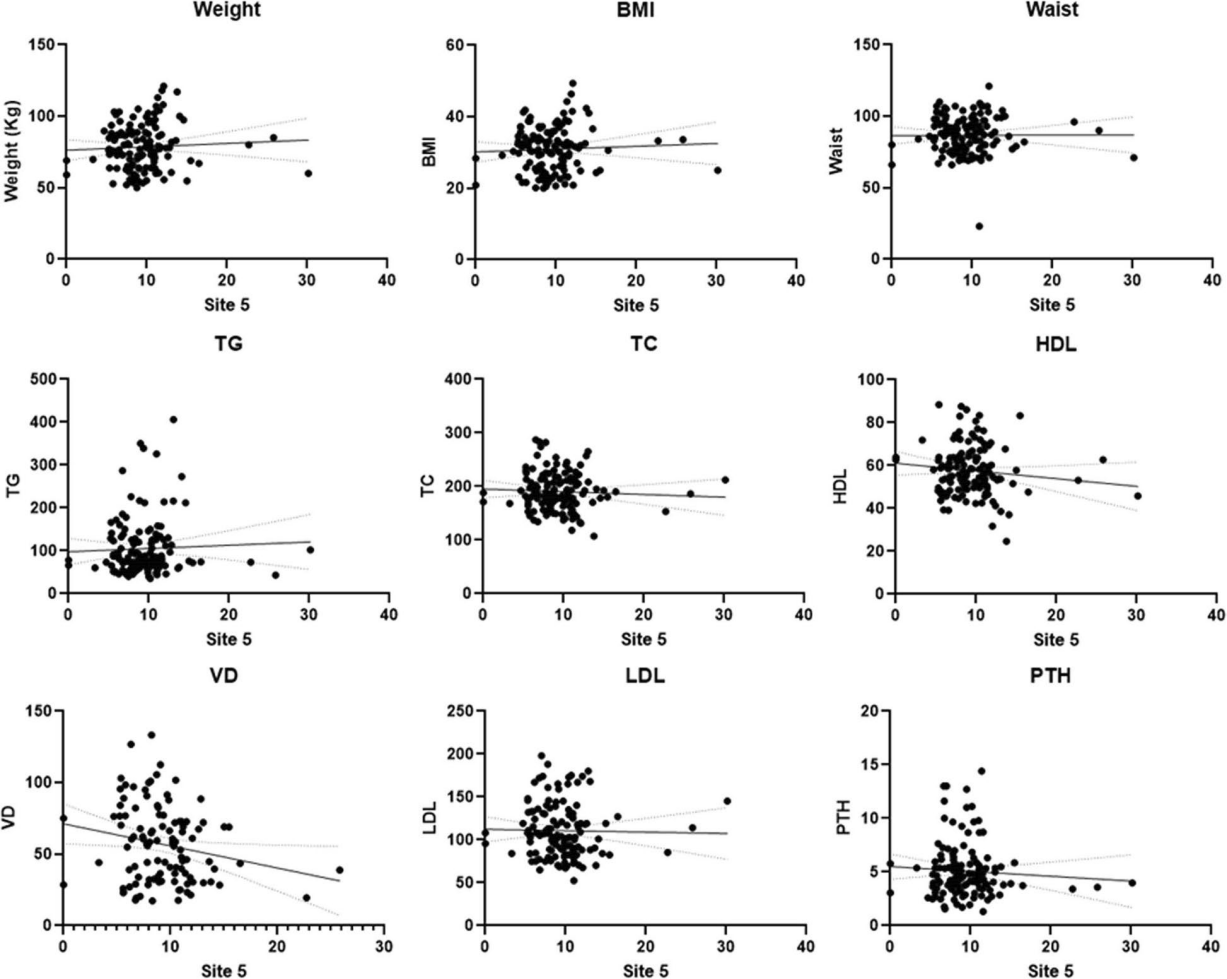
Spearman correlations between site 5 of CYP27B1 methylations with different anthropometric and biochemical parameters

These data indicate the direct and/or indirect role of CYP2R1 and CYP27B1 in obesity patients in this study. Spearman’s correlation coefficient clearly reveals a significant correlation between hypermethylation CpG sites of CYP2R1 and BMI, body weight, waist circumference, TC, LDL, and VD (at close to borderline significance *p* > 0.06) of obese patients (Figs. 4 and 5). However, no significant association of methylation of the five different sites in CYP27B1 with the lipid profiles was noticed. Also, the evidence from the best fit line curve of Spearman’s correlation coefficient graph indicates that the hypermethylation in the CpG site 5 of CYP27B1 significantly correlates with deficiency of vitamin D levels (Fig. 6). This is an important implication that CYP2R1 and CYP27B1 are crucial role players of vitamin D metabolism and pathophysiological mechanisms of obesity.

## Discussion

Epigenetic changes have been associated with the development of several chronic diseases including obesity and cancer [10, 20]. Analysis of methylation at the level of DNA provides information about gene regulation and identifies the potential markers of such chronic diseases [12]. Studies reported that irregular gene expression patterns due to epigenetic alterations and functions of VDR, CYP2R1, and CYP27B1 lead to specific target-associated pathophysiological conditions [1, 9]. Several reports have shown that epigenetic changes are associated with induction of obesity and alteration of vitamin D metabolism by altering the expression of crucial genes such as VDR, CYP2R1, and CYP27B1 and thereby influence the VD-associated metabolites and abnormal homeostasis of lipid profile [3, 21]. Our previous study [2] also clearly revealed that VDR methylation and SNPs influence the expression of CYP2R1 and CYP27B1 genes.

CYP2R1 and CYP27B1 genes are members of P450 superfamily enzymes which are directly involved in vitamin D metabolic pathways and also indirectly regulate synthesis of cholesterol, steroid, and various lipids [15, 18, 16]. Previous studies have expounded the importance of vitamin D in adipose physiology. Particularly, CYP2R1 and CYP27B1 genes are involved in adipose tissue homeostasis [11]. Taken together, these results indicate a positive correlation between obese and nonobese individuals, and that CYP2R1 and CYP27B1 genes are involved in lipid metabolism. Moreover, these data are consistent with our previous research wherein epigenetic changes and SNPs in the VDR gene have a strong correlation with anthropometric parameters such as body weight, BMI, waist circumference, and lipid profile in obese patients [2]. Bakos et al. [4] have reported that SNPs in CYP2R1 gene are strongly associated with severely vitamin D-deficient individuals. In another study, mutated CYP2R1 gene has been shown as linked to obesity and type 2 diabetes mellitus [31]. In a meta-analysis study, CYP2R1 and CYP27B1 genes were shown linked with different anthropometric measures of obesity and vitamin D deficiency patients [17]. In a similar study, CYP2R1 gene variants are shown as connected to BMI and body weight [7]. In a Finnish study, genetic variants of CYP2R1 gene were linked to total and LDL cholesterols [24], which confirms the findings in our study.

## Conclusions

The objective of this study was to determine the association between obesity and methylation of vitamin D-dependent genes (i.e., CYP2R1 and CYP27B1). Vitamin D-specific downstream targets with risk of obesity-related parameters including height, weight, BMI, 25-hydroxyvitamin D, parathyroid hormone, glycemic status, and lipid profile (TG, LDL, HDL, and TC) have provided strong clues to clarify the molecular mechanisms. These genes underlie the pathogenesis of vitamin D-associated diseases and may have potential application in the clinical diagnosis and treatment of obesity-linked diseases in the future.

## Supplementary Information

**Additional file 1: Table S1.** Comparison between total promoter methylation of CYP27B1 of normal and obese samples. **Fig. S1** Comparison between total promoter methylation of CYP27B1 of normal and obese samples. **Fig. S2.** Spearman Correlations between site 1 of CYP2R1 methylations with different anthropometric and biochemical parameters. **Fig. S3.** Spearman Correlations between site 3 of CYP2R1 methylations with different anthropometric and biochemical parameters. **Fig. S4.** Spearman Correlations between site 1 of CYP27B1 methylations with different anthropometric and biochemical parameters. **Fig. S5.** Spearman Correlations between site 2 of CYP27B1 methylations with different anthropometric and biochemical parameters. **Fig. S6.** Spearman Correlations between site 3 of CYP27B1 methylations with different anthropometric and biochemical parameters. **Fig. S7.** Spearman Correlations between site 4 of CYP27B1 methylations with different anthropometric and biochemical parameters.
