# Supplementary material for: Association of CYP2R1 and CYP27B1 genes with the risk of obesity and vitamin D metabolism in Saudi women
Source: J Genet Eng Biotechnol. 2023 May 15;21:59. doi: 10.1186/s43141-023-00508-7 (PMC10185724; doi:10.1186/s43141-023-00508-7)

**Supplementary data**

Table S1: Comparison between total promoter methylation of CYP27B1 of normal and obese samples

| **Parameters** | **Groups** | **N** | **Min.** | **Max.** | **Mean ± SD** | **Percent change** | **P Value** |
| --- | --- | --- | --- | --- | --- | --- | --- |
| Site 1^a^ | Normal | 39 | 0.00 | 18.57 | 10.62±3.70 | 100.00 | 0.732 |
|  | Obese | 92 | 0.00 | 17.98 | 10.80±3.78 | 101.78 |  |
| Site 2^a^ | Normal | 39 | 0.00 | 5.09 | 2.64±1.16 | 100.00 | 0.970 |
|  | Obese | 92 | 0.00 | 5.17 | 2.64±1.08 | 99.84 |  |
| Site 3^a^ | Normal | 39 | 6.58 | 31.78 | 12.77±4.57 | 100.00 | 0.285 |
|  | Obese | 92 | 4.01 | 25.67 | 13.26±4.27 | 103.84 |  |
| Site 4^a^ | Normal | 39 | 0.00 | 4.95 | 2.27±1.15 | 100.00 | 0.910 |
|  | Obese | 92 | 0.00 | 7.94 | 2.26±1.14 | 99.65 |  |
| Site 5^a^ | Normal | 39 | 0.00 | 30.15 | 9.60±4.30 | 100.00 | 0.902 |
|  | Obese | 92 | 0.00 | 25.82 | 9.36±3.56 | 97.53 |  |
| Total^b^ | Normal | 39 | 14.92 | 67.02 | 37.89±11.21 | 100.00 | 0.846 |
|  | Obese | 92 | 11.39 | 68.68 | 38.32±11.67 | 101.14 |  |

^a^ Comparison between groups using Mann-Whitney Test (Nonparametric data)

^b^ Comparison between groups using Independent samples T- Test (Nonparametric data)

**Figure S1 :** Comparison between total promoter methylation of CYP27B1 of normal and obese samples.


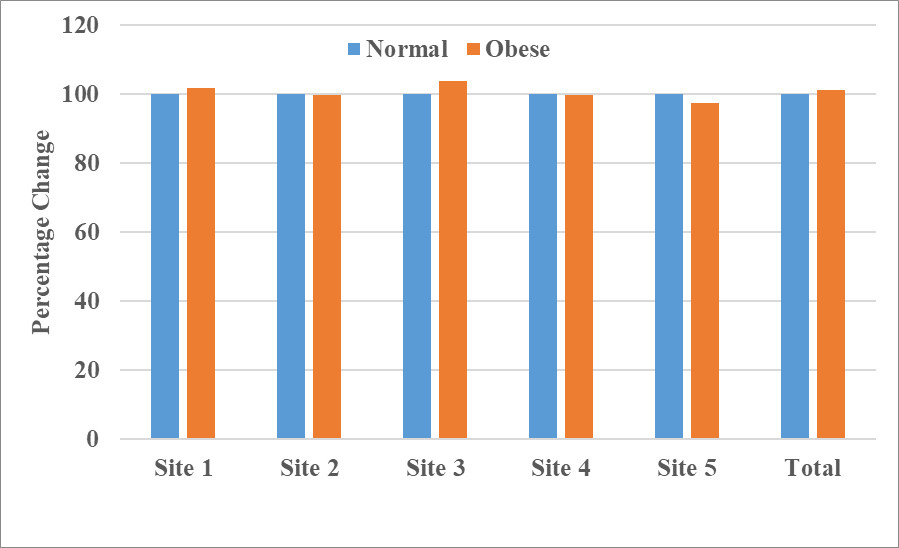


**Figure S2:** Spearman Correlations between site 1 of CYP2R1 methylations with different anthropometric and biochemical parameters.

**
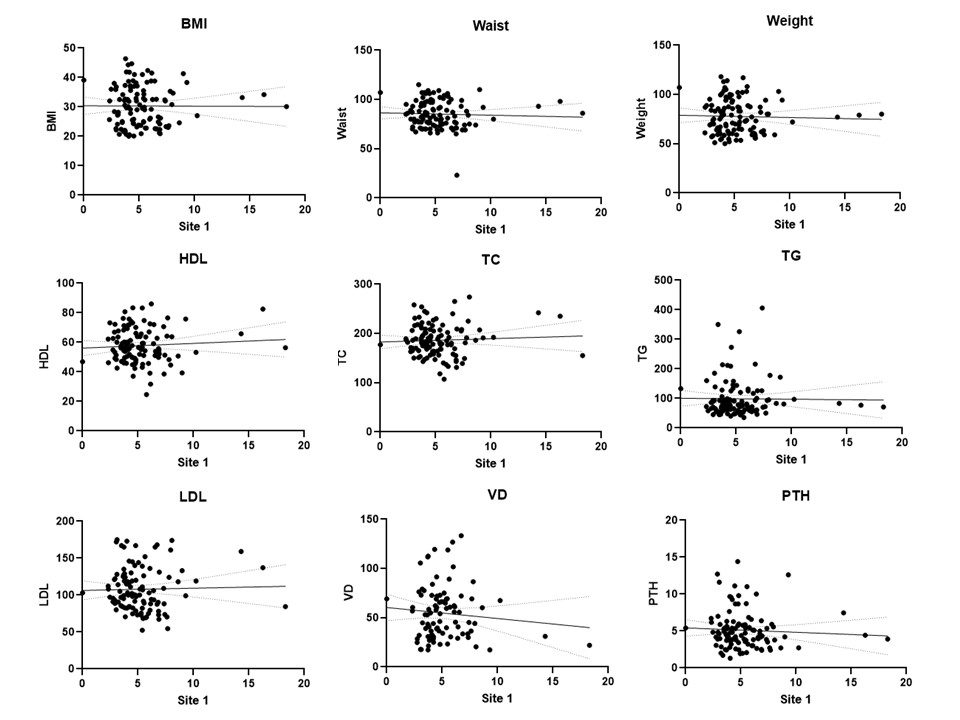
**

**Figure S3:** Spearman Correlations between site 3 of CYP2R1 methylations with different anthropometric and biochemical parameters.

**
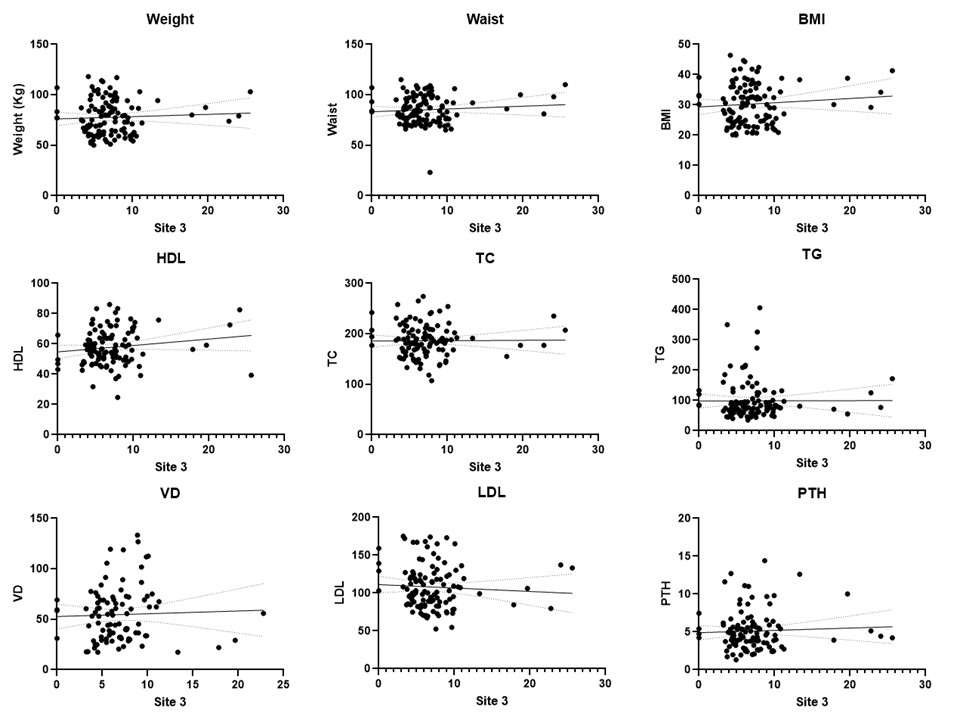
**

**Figure S4:** Spearman Correlations between site 1 of CYP27B1 methylations with different anthropometric and biochemical parameters.


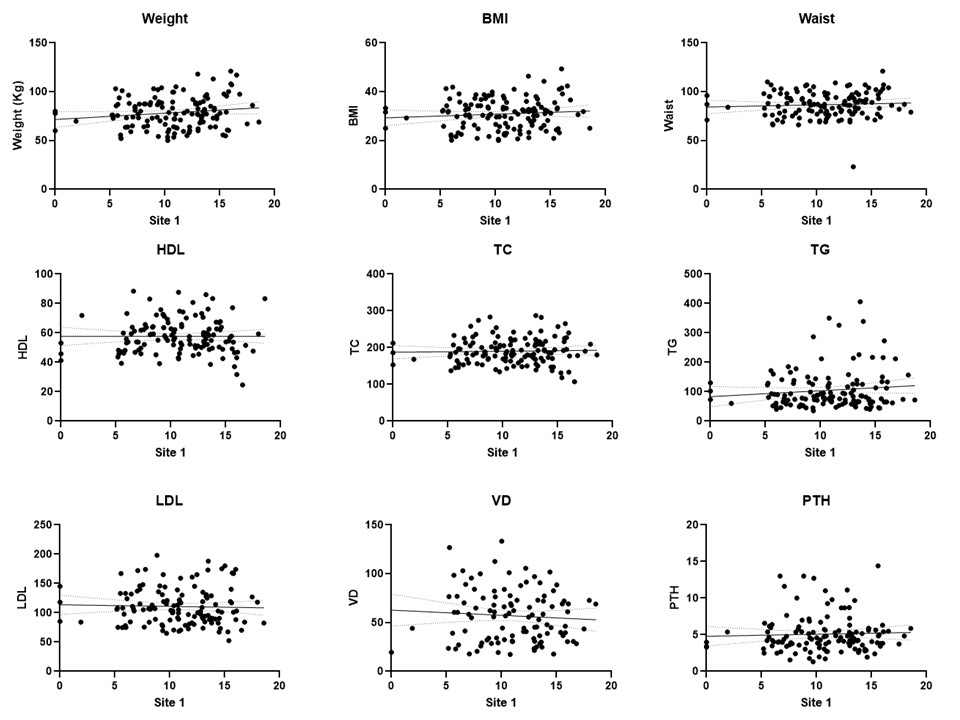


**Figure S5:** Spearman Correlations between site 2 of CYP27B1 methylations with different anthropometric and biochemical parameters.

**
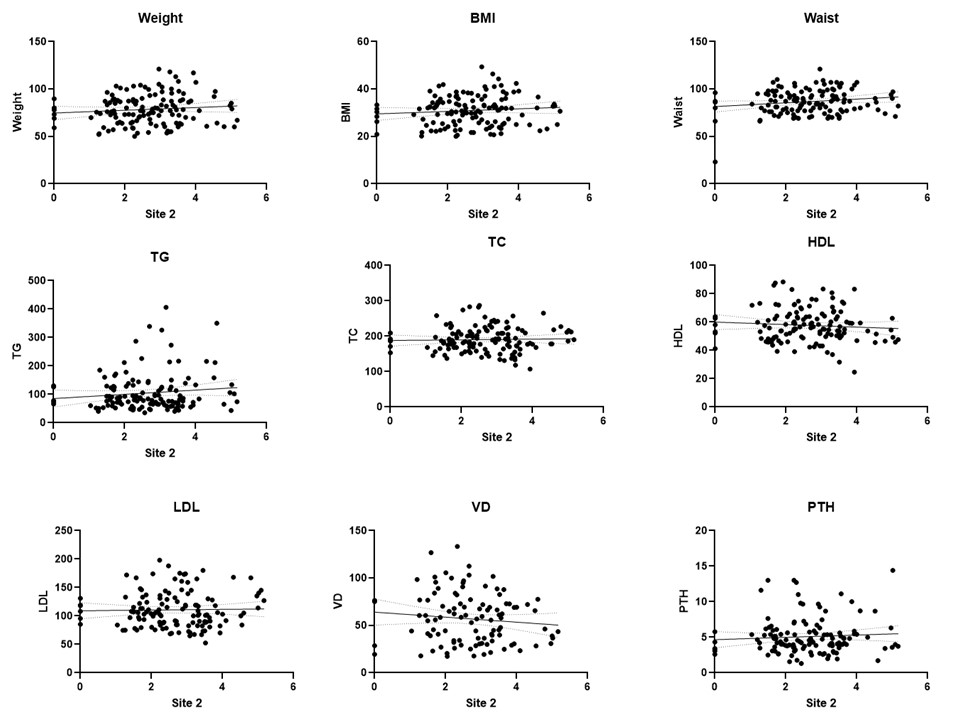
**

**Figure S6:** Spearman Correlations between site 3 of CYP27B1 methylations with different anthropometric and biochemical parameters.

**
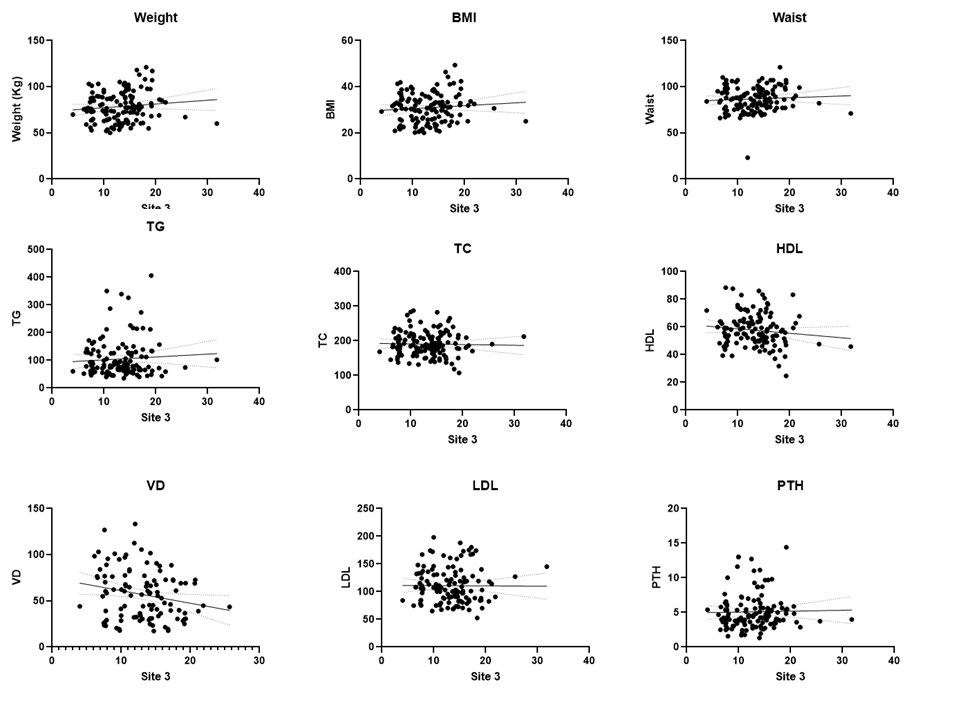
**

**Figure S7:** Spearman Correlations between site 4 of CYP27B1 methylations with different anthropometric and biochemical parameters.


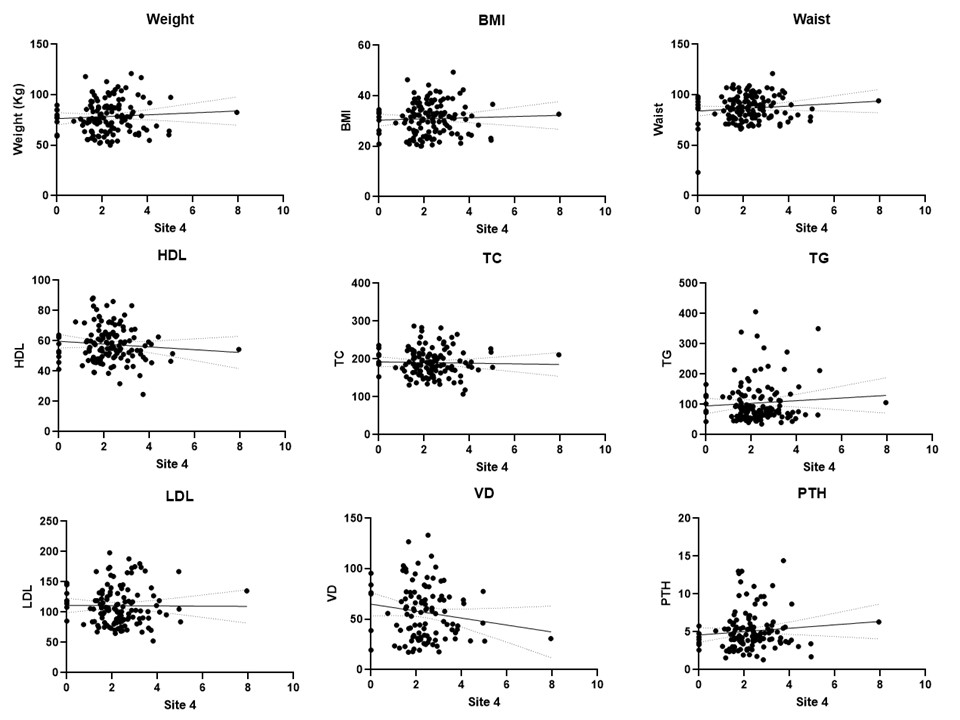

Supplement: Supplementary file 1 — Additional file 1: Table S1. Comparison between total promoter methylation of CYP27B1 of normal and obese samples. Fig. S1 Comparison between total promoter methylation of CYP27B1 of normal and obese samples. Fig. S2. Spearman Correlations between site 1 of CYP2R1 methylations with different anthropometric and biochemical parameters. Fig. S3. Spearman Correlations between site 3 of CYP2R1 methylations with different anthropometric and biochemical parameters. Fig. S4. Spearman Correlations between site 1 of CYP27B1 methylations with different anthropometric and biochemical parameters. Fig. S5. Spearman Correlations between site 2 of CYP27B1 methylations with different anthropometric and biochemical parameters. Fig. S6. Spearman Correlations between site 3 of CYP27B1 methylations with different anthropometric and biochemical parameters. Fig. S7. Spearman Correlations between site 4 of CYP27B1 methylations with different anthropometric and biochemical parameters. [file 43141_2023_508_MOESM1_ESM.docx]
